# A biofunctional review of C-reactive protein (CRP) as a mediator of inflammatory and immune responses: differentiating pentameric and modified CRP isoform effects

**DOI:** 10.3389/fimmu.2023.1264383

**Published:** 2023-09-15

**Authors:** Margaret E. Olson, Mary G. Hornick, Ashley Stefanski, Haya R. Albanna, Alesia Gjoni, Griffin D. Hall, Peter C. Hart, Ibraheem M. Rajab, Lawrence A. Potempa

**Affiliations:** College of Science, Health and Pharmacy, Roosevelt University, Schaumburg, IL, United States

**Keywords:** CRP isoforms, mCRP, inflammation, C-reactive protein, innate immunity, complement

## Abstract

C-reactive protein (CRP) is an acute phase, predominantly hepatically synthesized protein, secreted in response to cytokine signaling at sites of tissue injury or infection with the physiological function of acute pro-inflammatory response. Historically, CRP has been classified as a mediator of the innate immune system, acting as a pattern recognition receptor for phosphocholine-containing ligands. For decades, CRP was envisioned as a single, non-glycosylated, multi-subunit protein arranged non-covalently in cyclic symmetry around a central void. Over the past few years, however, CRP has been shown to exist in at least three distinct isoforms: 1.) a pentamer of five identical globular subunits (pCRP), 2.) a modified monomer (mCRP) resulting from a conformational change when subunits are dissociated from the pentamer, and 3.) a transitional isoform where the pentamer remains intact but is partially changed to express mCRP structural characteristics (referred to as pCRP* or mCRP_m_). The conversion of pCRP into mCRP can occur spontaneously and is observed under commonly used experimental conditions. In careful consideration of experimental design used in published reports of *in vitro* pro- and anti-inflammatory CRP bioactivities, we herein provide an interpretation of how distinctive CRP isoforms may have affected reported results. We argue that pro-inflammatory amplification mechanisms are consistent with the biofunction of mCRP, while weak anti-inflammatory mechanisms are consistent with pCRP. The interplay of each CRP isoform with specific immune cells (platelets, neutrophils, monocytes, endothelial cells, natural killer cells) and mechanisms of the innate immune system (complement), as well as differences in mCRP and pCRP ligand recognition and effector functions are discussed. This review will serve as a revised understanding of the structure-function relationship between CRP isoforms as related to inflammation and innate immunity mechanisms.

## Introduction

**Figure 1:**
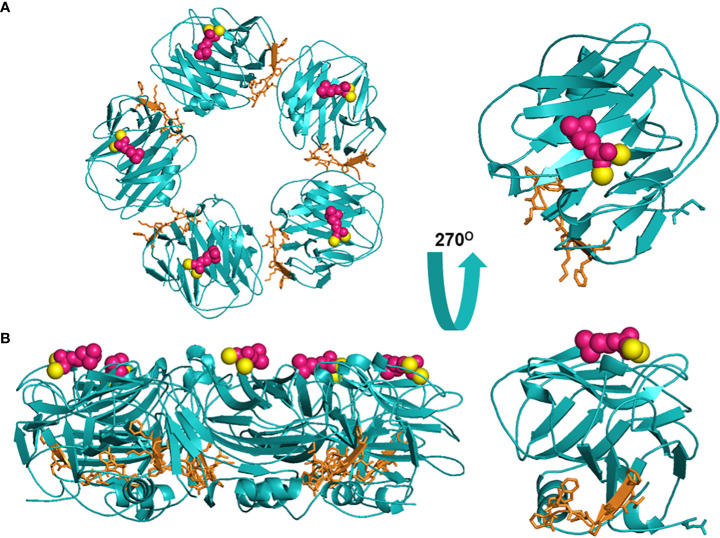
Structural rendering of pCRP and a pCRP monomer (PDB: 1B09). **(A)** provides a top-down view of pCRP and the pCRP monomer with the PC-binding face oriented upwards. **(B)** offers a side profile of pCRP and pCRP monomer. PC is depicted in pink, calcium ions in yellow, and the neoepitope (aa199-206) in orange. The x-ray crystal structure illustrates the Ca^2+^-dependence of PC-pCRP binding, which can occur on each pCRP subunit. pCRP^*^ binding to C1q occurs on the effector face, opposite (facing down) to the PC-binding face. Both views highlight how the neoepitope (orange) is buried within pCRP at the monomeric interfaces. While the present depiction of the pCRP monomer does not accurately represent mCRP structure given the secondary and tertiary changes that occur upon dissociation, release of mCRP clearly exposes the pro-inflammatory neoepitope for interaction with immune effector cells. Graphic was generated using PyMOL.

Despite long-standing recognition of C-reactive protein (CRP) as a non-specific, diagnostic biomarker for inflammation, only recently has the active role of CRP as an innate immune inflammatory mediator been revealed (1). CRP is primarily synthesized in the liver, though extrahepatic production in macrophages, vascular cells, endothelial cells, adipocytes, peripheral blood mononuclear cells (PBMCs), and the kidneys has been reported (2–11). The confounding functions of CRP relate to its dynamic, macromolecular structure in which CRP can exist in three isoforms: pCRP, pCRP*, and mCRP. Originally, “CRP”, oft referred to as nCRP (native/natural), was characterized as a 115 kDa pentamer of homologous subunits that are 206 amino acids in length (23 kDa each) (**Figure 1**) (1). The pentameric subunits self-assemble into a discoid tertiary structure via non-covalent bonding around a central pore (12). Pentameric structural integrity is stabilized by two calcium ions that also dictate CRP’s ligand binding capacity to phosphocholine (PC)-containing polysaccharides, polycations, chromatin, histone, ribonucleoprotein, fibronectin, laminin, lipoproteins, and galactins (12). It is now known that CRP acts as an agglutinin, opsonin and neutralizing protein that scavenges for debris at sites of active inflammation; yet in early CRP literature, several contradictory reports were made regarding the biofunctional properties of distinct CRP preparations. As early as the 1980’s, denatured CRP, via heat (H-CRP), metal chelation (F-CRP), acidic treatment (A-CRP), latex adsorption or freeze-thaw, yielded protein aggregates with antigenicity altered from native CRP (nCRP, primarily pCRP). The aforementioned conditions are now known to promote pCRP → pCRP^*^ → mCRP dissociation, exposing a neoepitope that is proinflammatory in nature (13–15). Subsequent studies demonstrated the *in vivo* relevance of this dissociation, which is prompted by acidic environments of inflammation in the body and PC/lipid ligand binding. Physiologically, CRP’s pro-inflammatory properties are evidenced by the presence of anti-(m)CRP antibodies in several autoimmune conditions, such as lupus nephritis, peripheral artery disease (PAD), and skin-related disorders (16–18). While a plethora of studies have now been performed to characterize the unique properties of the CRP isoforms, the field is absent of a single resource that reinterprets early CRP findings considering our current understanding. For example, initial investigation into CRP’s role as an inflammatory mediator were conducted with commercial CRP antisera, which is now known to contain recognition for both pCRP and mCRP, with up to 16% of the more potent mCRP neoepitope antigenicity present (19). The current review serves as the first comprehensive, detailed reevaluation of the historical CRP literature as related to immune system interactors with a current understanding of CRP conformational dynamics (**Table 1**). In the following studies, where a specific CRP isoform was evaluated, the pCRP, pCRP* and mCRP designators will be utilized. A study describing “CRP” should be assumed to refer to a biologically relevant mixture of CRP isoforms.

**Table 1:** Historically reported bioactivities of CRP vs. current understanding of mCRP.

Effector Response	Bioactivities of “CRP”^#^ 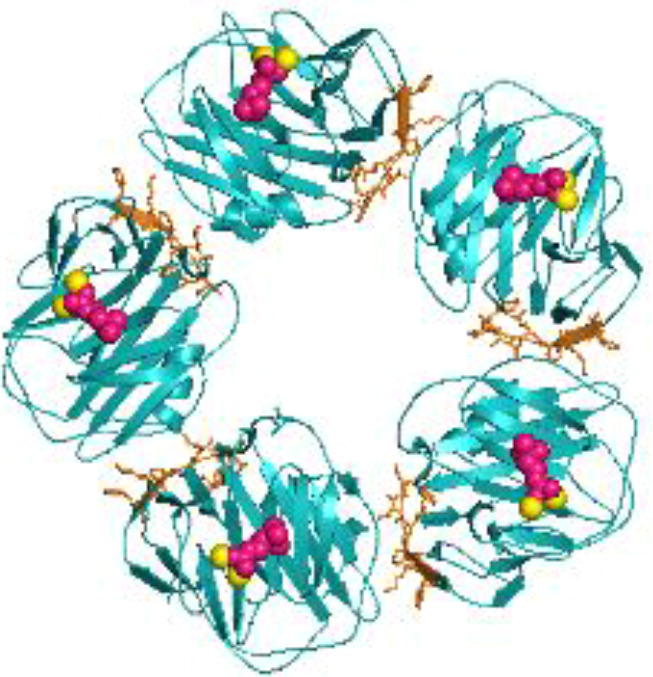	Bioactivities of mCRP 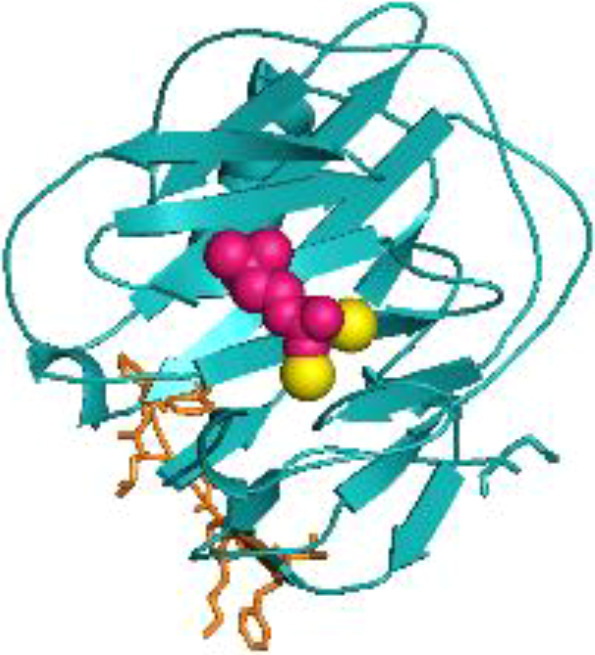
Platelets	• Maximizes responsiveness to ADP, epinephrine, thrombin, and collagen (38). • Promotes aggregation and secretion of dense and alpha granules (41). • Inhibition of PAF-induced platelet aggregation, activation, and platelet-neutrophil adherence (40). • Inhibition of arachidonic acid synthesis by phospholipase A_2_ (40).	• Stimulates/augments aggregation and secretion reactions (42, 43). • Upregulates P-selectin (44). • Increases prothrombotic activities under sheer conditions (44). • Captures and activates neutrophils (44).
Neutrophils	• Stimulates phagocytosis (45, 48–51). • Stimulates oxidative metabolism (41, 46, 55). • Inhibition of neutrophil activation and chemotaxis (45, 46). • Inhibition of ROS generation at higher concentrations (46, 52–54).	• Induces neutrophil trafficking (46, 57). • Reduces expression of L-selectin (65). • Increases adhesion to ECs (32, 66–68). • Stimulates IL-8 synthesis and release (24, 56). • Stimulates iNOS-mediated NO synthesis (20, 64). • Increases superoxide and peroxynitrite formation (ONOO^-^)(56). • Increases NF-κB and AP-1 (56). • Increases neutrophil-neutrophil and neutrophil-endothelial cell aggregation (44, 65, 66).
Monocytes	• Activates monocytes for phagocytosis (58, 69–71). • Stimulates oxidative responses (41). • Induces TF synthesis (62). • Promotes release of IL-1α, IL-1β, IL-6, IL-8, TNF-α, GRO-alpha and GRO-beta (60–62). • Upregulates liver X receptor-alpha (63).	• Potentiates respiratory burst response and increases ROS (41). • Stimulates cytokine release (60–62). • Increases monocyte adherence to ECs (32, 66–68). • Increases production of NO by increasing iNOS levels (20, 64).
Natural Killer Cells		• Enhances NK function (19, 72–75).
Endothelial Cells	• Induces EC adhesion (32, 66–68). • Inhibits eNOS (67).	• Increases MCP-1, E-selectin, and IL-8 expression (25). • Increases EC adhesion via ICAM-1 and VCAM-1 (44, 67, 76).
Complement	• Regulates C activation (49, 69, 77–84). • Binds C1q (85). • Enhances opsonization for phagocytosis (50, 70, 71, 86).	• Binds C1q when complexed with LDL. Does not activate C in fluid phase (85). • Binds Factor H and C4bp (34, 87). • Binds properdin (88).

Bullet points colored in blue list anti-inflammatory CRP properties now known to be pCRP-specific.

^#^The designation “CRP” was historically understood to be pCRP, but likely reflects a mixture of pCRP and mCRP bioactivity.

## Posited pCRP → mCRP conformational dynamics

In addition to their distinctive biofunctional features, CRP isoforms have unique pharmacokinetic properties, where pCRP is sera soluble. Conversely, mCRP is insoluble in sera unless protein-bound and is predominantly membrane-bound. pCRP, due to its detectability via blood testing, is the non-specific, diagnostic biomarker of inflammation, oft described in the literature, which rapidly rises up to 1000-fold in response to acute infection or injury (20). pCRP is also utilized as a prognostic marker for diseases of chronic inflammation, including autoimmune and cardiovascular diseases (1). Circulating pCRP localizes to sites of damaged tissue (infection/injury) prior to undergoing a conformational change upon binding to damaged membranes, which exposes the pro-inflammatory CRP neoepitope of amino acids 199-206 (21, 22). Binding of pCRP to damaged membranes is posited to occur via lysophosphatidylcholine (LPC), a bioactive lipid found on the immune cell surface (23–25). Direct binding between the neoepitope of pCRP*/mCRP and membrane receptors is suggested to occur via FcγRIIIa (CD16), but this binding event only partially explains mCRP’s adhesive properties. Hydrophobic interactions with lipid rafts are predicted to compensate for non-receptor binding. Evidence for lipid-promoted conformational dynamics is exhibited by the association of mCRP with extracellular lipid vesicles (26). Activated leukocytes slough membrane-bound CRP, which enters circulation as extracellular vesicles upon cleavage. In an *in vitro* study, Trail et al. demonstrated lipid microparticle-promoted pCRP to mCRP dissociation on a timescale of twenty minutes (27). *In vivo*, mCRP-linked lipid microparticles are observed in PAD and after myocardial infarction with pro-inflammatory function (16, 28). Initially, pCRP transitions to pCRP*, a transient conformation that maintains a pentameric quaternary structure but displays the neoepitope of mCRP (21, 29, 30). pCRP* further dissociates to proinflammatory mCRP (31). pCRP is completely converted to mCRP in 24-48 hrs (21). mCRP can also be generated *in vitro* in denaturing (heat, acid, chelation) or oxidative environments; conditions that were initially reported to generate an antigenic, serum-insoluble aggregate (13). mCRP exhibits the greatest proinflammatory activity once the intrachain disulfide bond, which staples together the two antiparallel β-sheets of the CRP monomer, is reduced (22). pCRP and mCRP isoforms can now be distinguished with conformation-specific antibodies (9C9, 3H12), though these antibodies are unable to parse pCRP* from mCRP (32–37). Importantly, the pCRP → pCRP* → mCRP transition is unidirectional, with mCRP incapable of regenerating pCRP.

## mCRP promotes platelet aggregation

Early studies investigating the effects of H-CRP, a thermally aggregated form, on platelet function demonstrated that H-CRP stimulated platelet aggregation under isolated, *in vitro* conditions, similar to heat-treated human IgG, and promoted the corresponding secretion of adenosine triphosphate (ATP) and β-thromboglobulin (38). While determined *not* to be a direct platelet agonist in the more complex environment of platelet-rich plasma (PRP), synergism between H-CRP and adenosine diphosphate (ADP) was observed resulting in irreversible platelet aggregation and ATP secretion. In an optimized combination, mCRP and ADP promoted the release of secretory granule components. H-CRP also synergized with epinephrine, collagen, and thrombin. Interestingly, H-CRP was produced by heat treatment at 63 ^o^C, a temperature now known to promote pCRP to mCRP dissociation. The authors note that heat treatment forced aqueous insoluble protein aggregates accounting for 47-53% of the total CRP preparation. Upon removal of the initial H-CRP aggregate, an additional ~50% CRP could be precipitated with a second heat treatment. Both lots of H-CRP were effective in synergizing with known platelet activators in PRP. Given the heat dependence of CRP aggregation and their insoluble properties in aqueous buffer, it makes sense that the observed aggregates were enriched for mCRP, and the platelet activating properties are inherent to this isoform. Moreover, unmodified CRP, without heat treatment, failed to demonstrate platelet activating potential (39).

Conversely, several concurrent studies reported a protective role for CRP against platelet aggregation (40). Specifically, CRP, of unreported conformation, inhibited platelet-activating factor (PAF)-induced platelet aggregation in a dose dependent manner (1-20 µg/ml) (40). This CRP preparation also inhibited the synthesis of arachidonic acid by phospholipase A_2_, limiting the production of proinflammatory cytokines. While no methodology is included to describe CRP’s preparation in this study, it is reasonable to assume that the CRP was primarily unmodified. If so, these contradictory results to the former studies may be rationalized by the majority presence of pCRP.

In addition to heat aggregated CRP (H-CRP), other chemically-modified CRPs, including urea chelated (F), latex adsorbed, and acid (A)-aggregated, have been found to activate platelet aggregation, promote dense and alpha-granule secretion and upregulate thromboxane A2 synthesis (41). Treatment of CRP with hypochlorous acid (HOCl) prompted oxidative protein unfolding, exposing the hydrophobic interior of CRP in a tertiary structure analogous to mCRP (42). HOCl-CRP (50 µg/ml) activated platelets in isolated preparations, inducing aggregation by ~80%, and was shown to interact with several platelet receptors (TLR-4, GPIIbIIIa) and plasma proteins (C1q, IgG). Like with heat treatment, these observations suggest that acidic environments can promote pCRP to mCRP dissociation, which describes CRP’s platelet activating properties. Importantly, a pH between 6-7 is commonly found at sites of inflammation. Further support for oxidative-mediated dissociation of pCRP to mCRP was observed when nCRP (pCRP) failed to activate platelets, though CRP treated with reactive oxygen species (ROS: Fe^2+^ - Cu^2+^ - ascorbate) irreversibly activated platelets in the presence of suboptimal levels of PAF, thrombin and ADP (43). Li et al. subsequently reported that Cu^2+^-induced oxidative and acidic environments induce the pCRP → mCRP isoform transition (17). Taken together, when CRP is properly activated, i.e., in the mCRP conformation, platelet aggregation and activation is augmented. When later tested with recombinant proteins, mCRP activated and pCRP inhibited pro-thrombotic activities under sheer conditions (44).

## mCRP stimulates neutrophil migration, chemotaxis & phagocytosis; pCRP inhibits neutrophil activation and adhesion

Early studies of CRP effects on neutrophil function probed CRP uptake as a means of pathogenic ligand clearance and found that CRP enhanced neutrophil-mediated clearance of several bacterial species via binding interactions with the PC-containing bacterial membrane (45). In one study, CRP was found to mediate pneumococcal C-polysaccharide (CPS) uptake into neutrophils, promoting clearance, particularly in combination with activated complement (C). This CRP preparation was found to have no effect on neutrophil migration, leukotaxis, oxidative metabolism, or chemiluminescence. The uptake of CPS was CRP-dependent, as CPS alone was not phagocytosed by neutrophils. Though not as efficient, non-complexed CRP was internalized by neutrophils, while uptake was enhanced with the CRP-CPS complex. As CRP binding to PC-containing CPS promotes the pCRP to pCRP^*^ transition, a change that displays structural and antigenic characteristics of mCRP, the ultimate adhesive effects are characteristic of pro-inflammatory mCRP function. Interestingly, non-complexed CRP uptake was reduced in the presence of EDTA, which preferences the mCRP isoform. Interpreting these findings considering current CRP conformational dynamics, it is likely that pentameric forms of CRP must initiate ligand binding before CRP fully dissociates to mCRP.

Subsequent studies demonstrated the concentration dependence of CRP’s effects on neutrophils. Low CRP concentrations of 0.1-1 μg/mL *do* promote neutrophil chemotaxis, but this effect is lost at higher concentrations (46). The abovementioned study interrogated neutrophil chemotaxis at significantly higher CRP concentrations of 60-120 μg/mL, where no effect on leukotaxis was observed. In further studies, CRP concentrations of 25 μg/mL demonstrated dose-dependent *inhibition* of neutrophil migration towards chemotactic stimuli with complete inhibition at 100 μg/mL. CRP-specific leukotaxis could be explained by mCRP-specific receptors on the neutrophil surface and/or through mCRP-neutrophil hydrophobic interactions. Given the hydrophobic properties of mCRP, low CRP concentrations may preferentially represent the mCRP isoform due to rapid conversion of pCRP → mCRP, exposing a hydrophobic binding surface for neutrophil adhesion. The role of hydrophobic binding interactions in chemotaxis has been described (47). Importantly, the binding interaction between CRP and neutrophils is saturable at CRP concentrations of <5 μg/mL, a range applicable to the observed activating effects of mCRP on neutrophils (46). Putatively, after mCRP binding sites are saturated, higher CRP concentrations, with increased pCRP representation, could promote inhibitory effects. This hypothesis parallels the acute inflammatory response where the majority of pCRP present at the earliest phases of host defense will be converted to mCRP (31).

A concentration dependent effect of CRP in combination with phorbol 12-myristate 13-acetate (PMA) on superoxide anion generation and secretion was also observed, where CRP <5 µg/mL demonstrated potentiation but CRP >10 µg/ml caused inhibition of this response (46). ROS generation results from the phagocytic respiratory burst of neutrophils and can be measured by light emission (chemiluminescence). At the acute phase CRP concentration of 1 μg/mL, CRP enhanced neutrophil phagocytosis, even of non-PC liganded particles, extending the previous findings that CRP promoted neutrophil opsonization of PC-containing bacterial membranes/capsules (48–51). These findings are further supported by the observation that CRP inhibits PAF-mediated chemiluminescence at higher concentrations of 100 μg/mL (52). The concentration dependence of these studies may allude to the conformational dynamics of pCRP → mCRP transition, though this hypothesis is limited by the inability of mCRP to reassemble to pCRP. An alternative explanation is that high concentrations of CRP (>10 µg/mL) increase intracellular cAMP levels; high cAMP concentrations are known to inhibit degranulation and PMA-activated neutrophil oxidative metabolism (53, 54). It is important to note that the cAMP-raising concentration of CRP at >10 µg/mL is greater than CRP concentrations with neutrophil inhibitory effects. Therefore, a relationship may exist where low CRP concentrations, inclusive of mCRP, exhibit proinflammatory function (increased adhesion/chemotaxis) to maximize damage to infective agents while simultaneously raising cAMP levels. At a certain threshold, both CRP and cAMP concentrations rise to a level with anti-inflammatory properties, putatively to minimize damage to the host.

In further studies with H-CRP, which is preferenced for mCRP, chemiluminescence potentiation between H-CRP and heat aggregated-IgG (H-IgG) is observed (55). H-CRP was unable to stimulate hydrogen peroxide generation in neutrophils alone, but when dosed with H-IgG, hydrogen peroxide generation was significantly enhanced. Importantly, this potentiation was selectively limited to intracellular H_2_O_2_ generation, and failed to upregulate ROS release from neutrophils, putatively describing a pathogen-fighting mechanism within neutrophils at infected sites that limits ROS damage to surrounding tissues. Later studies demonstrated that F- and A-CRP similarly potentiate the chemiluminescent output of H-IgG treated neutrophils (41). nCRP (pCRP) exhibited no increase in H-IgG-induced chemiluminescence. In these studies, F-CRP, A-CRP, and H-CRP were dosed as mixtures of aggregated and soluble protein, with the potentiating activity associated solely with the protein aggregates. This observation tracks with insoluble mCRP exhibiting the proinflammatory phagocytotic characteristics, while soluble pCRP does not.

More recently, the distinct effects of pCRP and mCRP on neutrophils have been characterized. Recombinant mCRP increased superoxide and peroxynitrite (ONOO^-^) production, subsequently causing a rise in nuclear factor κB (NF-κB) and activator protein-1 (AP-1) (56). pCRP had no corresponding effect on ROS production within a similar timeframe. As both ONOO^-^ and NF-κB are known to increase IL-8, the impact of recombinant mCRP on IL-8 concentration was evaluated and increased levels were observed within 4 hours. pCRP also upregulated IL-8 expression, but on a timescale of 24-hr, which suggests pCRP dissociated to mCRP (24). mCRP-mediated effects were nullified with an anti-CD16 antibody; pCRP does not bind CD16 while mCRP does. In addition to ROS generation, mCRP has also been implicated in neutrophil-platelet and neutrophil-neutrophil adhesion, a proinflammatory marker of poor cardiovascular outcomes (44). Conversely, preincubation of whole blood samples with pCRP decreased shear-induced neutrophil-platelet adhesion and neutrophil aggregation in a dose-dependent relationship. Treatment with H-CRP (*“mCRP”*) resulted in complete loss of this protective activity. mCRP’s proinflammatory effects are platelet P-selectin mediated, as mCRP enhanced P-selectin expression and increased the rate of neutrophil-platelet and neutrophil-neutrophil adducts in a dose dependent manner (1-50 µg/ml). The cellular adhesion molecule CD18 was also implicated. In a study of pCRP with anti-P-selectin and anti-CD18, nearly complete blockade of adhesion events was observed. pCRP effects were attenuated by anti-CD32, while mCRP effects were attenuated by anti-CD16.

In a similar vein, mCRP was found to drive tissue damage in ischemia/reperfusion injury via proinflammatory mechanisms of leukocyte-endothelial cell aggregation, leukotaxis, and ROS generation (57). These proinflammatory effects were successfully abrogated with the small molecule pCRP → mCRP dissociation inhibitor 1,6-bisPC.

## mCRP activates monocyte cytokine release

Monocytes regulate cellular homeostasis in times of inflammation by scavenging for invader cells and promoting the inflammatory response. Monocytes can differentiate into interstitial dendritic cells, microglial cells, and macrophages, and migrate into lesions inflamed by the immune system. As with neutrophils and platelets, the association of elevated serum CRP levels during the inflammatory response suggests a role for CRP in monocyte-macrophage infiltration (58). Prior to our understanding of distinct CRP isoforms, “CRP” (10 μg/mL) was found to induce macrophages to a tumoricidal state, (59) and CRP (50 μg/mL) prompted monocytes to release interleukin 1 beta (IL-1β), interleukin 6 (IL-6) and tumor necrosis factor alpha (TNF-α), with 10-fold higher levels of each cytokine observed at 4 hours (60, 61). Additional studies found CRP also promoted a 75-fold increase in tissue factor (TF)-mediated procoagulant activity, and significantly higher levels of interleukin-1alpha (IL-1α), GRO-alpha, GRO-beta and IL-8 (62). Interestingly, CRP also exhibited anti-inflammatory properties in the upregulation of liver X receptor (LXR)-alpha.(63)

Studies with H-CRP enabled the characterization of mCRP-specific activity; first for adhesion, 70% of monocytes and 8% of mononuclear leukocytes were found adhered to H-CRP (41). Moreover, a significant enhancement in chemiluminescence was observed in response to H-CRP/H-IgG treatment, resulting from the respiratory burst that occurs during monocyte activation. Thus, it is hypothesized that H-CRP synergizes with IgG to promote Fc receptor-mediated stimulation of monocyte oxidative metabolism. Recombinant mCRP was later demonstrated to increase nitric oxide (NO)/inducible nitric oxide synthase (iNOS) levels (64). Conversely, pCRP exhibited anti-inflammatory effects by decreasing NO/iNOS production in macrophages and iNOS activity in monocytes. As with neutrophils and platelets described previously, reevaluation of the historical literature presents an argument for CRP’s proinflammatory properties being inherent to the mCRP isoform, while pCRP exhibits the anti-inflammatory activity.

## mCRP upregulates endothelial cell adhesion

Proinflammatory effects associated with “CRP”, commercially available from Calbiochem, were also observed in early studies with umbilical vein and human coronary artery endothelial cells (HCAECs) (67, 76). Specifically, recombinant CRP upregulated intercellular adhesion molecules (ICAM-1) to levels nearly 10-fold baseline and increased vascular cell adhesion molecule (VCAM-1) and E-selectin. The induced expression mimicked the effects of IL-1β and is implicated in the recruitment of monocytes and leukocytes (89). CRP also exhibited an inhibitory effect on endothelial nitric oxide synthase (eNOS) at incubation times >24-hr, which has proinflammatory implications for atherogenesis (67). Reduced nitric oxide (NO) production leads to arterial vasoconstriction, platelet adhesion and aggregation, and monocyte-endothelium adhesion (32, 67, 68, 90). It was confirmed that in the presence of 24-hr “CRP” treatment, there was a significant increase in monocyte adhesion, VCAM-1, and ICAM-1 expression. Given the unknown storage conditions and age of the commercially available CRP, and the long incubation time of these experiments (24-hr), the presence of mCRP as a CRP sub-population is probable. Importantly, the *in vivo* half-life of pCRP is 19-24 hrs (91).

Once the existence of the mCRP isoform was elucidated, targeted studies on mCRP’s ability to activate endothelial cells were performed. mCRP (0.1–200 μg/mL), but not pCRP, was found to markedly upregulate CD11b/CD18 expression on neutrophils and enhance the adhesion of neutrophils to HCAECs (66). Adhesion was negated in the presence of an anti-CD18 mAb, correlating with the previous finding that pCRP with anti-P-selectin and anti-CD18 promoted complete blockade of adhesion between neutrophils and monocytes (See Neutrophil Discussion) (65). mCRP (1–30 μg/mL), specifically, also increased monocyte chemoattractant protein-1 (MCP-1) and IL-8 secretion, key mediators of leukocyte recruitment, as well as neutrophil-endothelial cell adhesion via the expression of ICAM-1, VCAM-1 and E-selectin (25). To observe the similar effects with nCRP preparations, a 6x longer incubation was required, implicating that pentameric dissociation had occurred.

Multiple studies implicated mitogen-activated protein kinase (MAPK) involvement in mCRP’s activation of endothelial cells as increased phosphorylation of p21^ras^ (RAS) and p38 MAPK, and downstream activation of Raf-1, MAPKK and ERK, was observed. As pCRP binds primarily to low-affinity IgG FcγRIIa (CD32) and somewhat to high affinity IgG FcγRI (CD64) while mCRP binds to low-affinity IgG immune complex FcγRIIIb (CD16) (92–94), the effect of anti-CD16 and anti-CD32 were interrogated. Anti-CD16, but not anti-CD32, reduced MCP-1 and IL-8 secretion, ICAM-1, VCAM-1 and E-selectin expression, and corresponding neutrophil-endothelial cell adhesion 14-32-fold. Moreover, these effects were blunted when mCRP was tested in the presence of p38 MAPK inhibitor SB 203580. The incomplete attenuation of anti-CD16 on mCRP’s proinflammatory effects was later explained by membrane insertion. mCRP also binds to cholesterol microdomains (lipid rafts) in membranes via aa35-47 (consensus cholesterol binding sequence) and aa199-206 (95). When mCRP’s ability to bind lipid rafts was inhibited by methyl-β-cyclodextrin or nystatin, MCP-1 and IL-8 secretion was also downregulated. Accordingly, adhesion between endothelial cells and neutrophils was also reduced.

## mCRP promotes natural killer cell activity

With physiologically relevant effects on effector cells of the innate immune response, the consequence of CRP on NK cell-mediated killing was explored starting in the early 1980’s. Specifically, the first experiments gauged whether NK cells bind CRP and whether such binding events affect NK function by treating human lymphocytes with anti-CRP and C (72). It was found that under such conditions, the ability of NK cells to kill cellular targets (K562, MOLT-4 cells) was significantly diminished. Using biotin-avidin amplification with biotinylated anti-CRP and fluorescent avidin, membrane-bound CRP specifically, and not plasma solubilized CRP, was determined to bind a subset of NK cells (~4%) and this binding event served as a promoter of NK activity (19). Like with previously discussed proinflammatory mechanisms, it is now understood that membrane (lipid raft)-bound CRP is enriched for the pCRP^*^/mCRP isoform and fluid-phase CRP is absent of the neoepitope (pCRP). Accordingly, the NK activating properties of CRP are associated with pentameric dissociation. Further refinement of this understanding comes from flow cytometry studies using α-neo-CRP antisera. In these studies, NK cells, displaying the surface markers CD16, CD11b, Leu-7 and Leu-19, reacted with the pCRP^*^/mCRP-recognizing antisera as did cells expressing B-cell markers (74). α-Native CRP failed to react with NK cells (73). Hamoudi and Baum further probed the mechanism of CRP-mediated NK activation by determining how anti-CRP inhibits NK cell lysis. As with previous studies, it was found that anti-CRP had no effect on the number of effector cell : target cell conjugates formed (75). Yet, the number of target cells killed by NK cells was largely diminished. This observation was negated when anti-CRP’s effect on NK cells was investigated in the absence of Ca^2+^. In the absence of Ca^2+^, pCRP dissociates to mCRP.

## CRP both activates and inhibits complement cascades

When CPS was added to serum containing nCRP, the components of the classical complement (C) pathway were consumed, suggesting that CRP-CPS complexation is essential to trigger CRP-mediated innate immune responses (77, 84). C activation was subsequently observed when CRP complexed with polycations, *i.e.*, protamine,(78–80) positively charged liposomes, (69) and nuclear DNA (83, 96). C activation resulting from CRP lipid binding requires either PC or sphingomyelin in a Ca^2+^-dependent manner (45, 77, 97–99). Lipid acyl chain length, degree of unsaturation, cholesterol concentration, and positively-charged lipid content (e.g. stearylamine, cetyltrimethylammonium, galactosyl ceramide) also impacted the degree of CRP binding (100). It is now known that ligand binding initiates conformational changes that expose the mCRP neoepitope facilitating binding to complement component 1q (C1q) and additional mediators of host defense. Ji et al. provided further evidence for this hypothesis by demonstrating that recombinant mCRP bound C1q in the C1q’s collagen-like region (85). Regarding bioactivity, mCRP bound to oxidized low-density lipoprotein (LDL) activated the classical C pathway upon C1q binding. Conversely, mCRP alone bound and inhibited C1q from engaging other complement intermediates.

CRP activates the classical C cascade by directly binding C1q at the membranes of damaged cells, leading to near complete consumption of C1, C4 and C2 and partial consumption of C3 with minimal activation of C5-C9 and no cell lysis (84, 86, 101). This favors opsonization without a strong, global inflammatory response. Activation of the C cascade by CRP stimulates monocyte and macrophage phagocytosis both *in vitro* and *in vivo* and enhances opsonization of microbes by phagocytic cells (50, 70, 71).

To limit inflammatory responses to the damaged area, CRP also influences alternative complement pathway (AP) activity, downregulating deposition of C3b on AP-activating surfaces, decreasing C3 and C5 convertase activity and inhibiting C amplification feedback (20). In particular, on stearylamine-containing liposomes and on Types 6 and R36a Streptococcal pneumonia surfaces, CRP decreased serum C3-activation implicating CRP as a regulatory protein that can limit the extent of inflammatory damage initiated by C activation (69, 102). Taken together, CRP binding to damaged membrane or bacterial surfaces can preference the immune response towards classical pathway activation and away from the AP (102). AP inhibitory activity was found to be Factor H dependent, with direct binding of Factor H to CRP implicated through ELISA experimentation. Factor H – CRP binding was not inhibited in the presence of EDTA or PC, highlighting the presence of pCRP in this inflammatory dampening response (71). Moreover, complement factor H-related protein, which is structurally similar to C Factor H, was demonstrated to bind both pCRP and mCRP, with the former binding event requiring Ca^2+^ (87). As CFHR4 localizes to necrotic tumor tissues and binds necrotic cells, CRP-CFHR4 binding is implicated in the opsonization of necrotic cells. CRP-mediated inhibition of AP activation can also be facilitated by C4b-binding protein (C4bp), which like Factor H, inhibits C cascade at the level of C3 (34). Interestingly, while evidence suggests that both mCRP and pCRP bind Factor H, C4bp binding is unique to mCRP to enhance degradation of C4b and C3b. Finally, mCRP was demonstrated to bind properdin, an initiator of the AP cascade that binds necrotic and ECs (88). pCRP failed to exhibit similar properdin binding. mCRP-bound properdin was unable to bind endothelial cells, thus inhibiting AP activation via C3 and C5b-9 deposition of ECs. In total, CRP acts as a regulator of C activation, recruiting both C1q, the activator, C4bp, the inhibitor of the classical pathway, and properdin, an inhibitor of the AP. As mCRP preferably binds both C4bp and properdin, the monomeric isoform likely elicits greater control over the C cascade. This design enables an acute, localized proinflammatory response that is subsequently inhibited to prevent further-reaching inflammatory damage.

## Conclusion

Reevaluating the historical CRP literature with a current understanding of distinct CRP isoforms clearly intimates both pro- and anti-inflammatory function as part of the acute immune response. Moreover, these isoform-defined activities help to explain the considerable differences in CRP expression, but similar phenotypes, across species (103, 104). While human CRP levels can rise to 1000-fold over baseline, mouse and rat CRP levels only rise 2-3-fold, respectively (105, 106). The disconnect between CRP expression levels and activity highlights the functional importance of the active mCRP isoform, in addition to the species’ mCRP generating capacity. Notably, rat CRP, which exists at baseline in the highest concentrations, exhibits the lowest mCRP generating capacity, while the inverse describes mice (low baseline levels, high mCRP generating capacity).(103, 104) Taken together, mCRP levels are responsible for the pro-inflammatory activities regardless of species.

**Figure 2:**
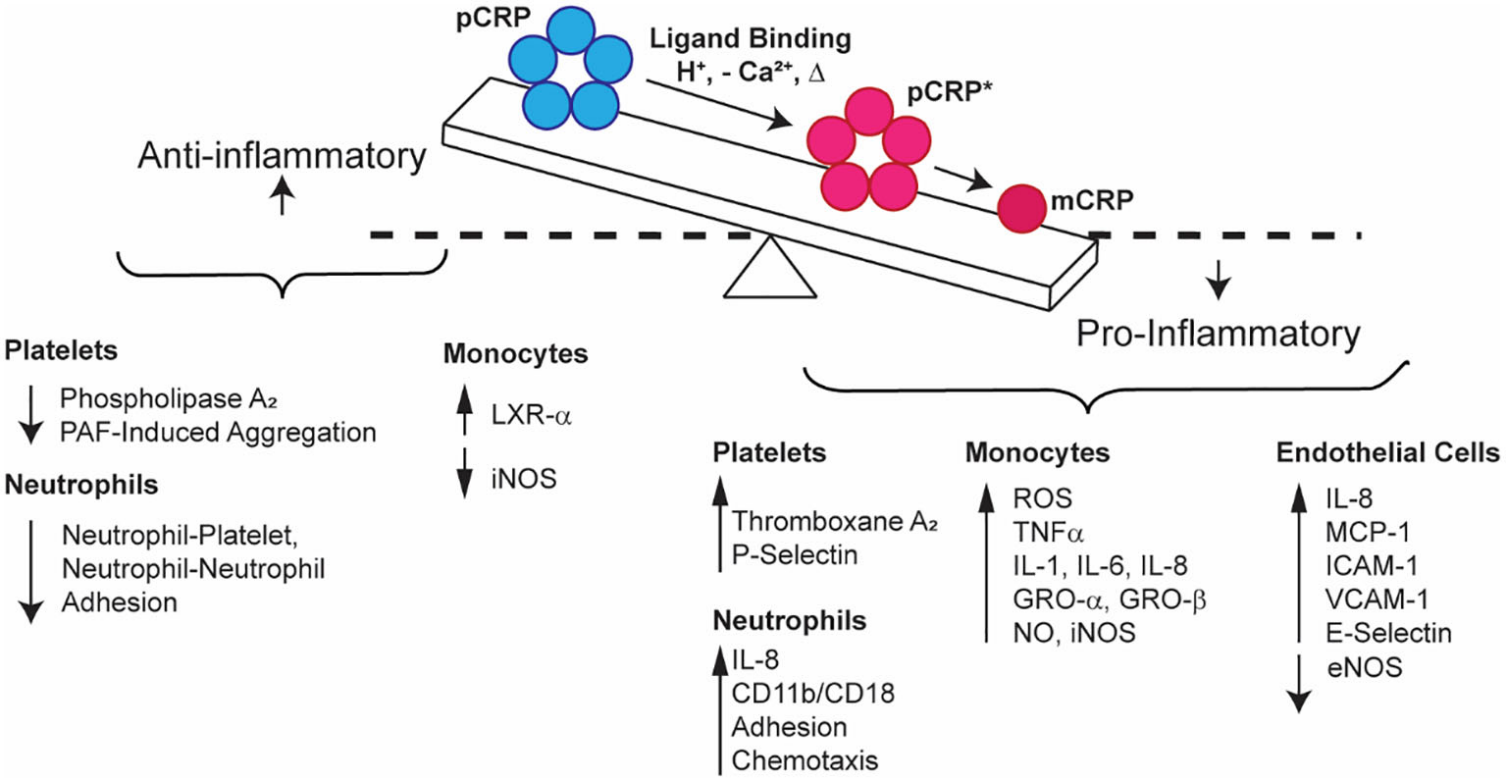
Schematic representation of the relationship between pro- (blue) and anti-inflammatory (red) CRP properties. pCRP exhibits anti-inflammatory outcomes, while mCRP exhibits proinflammatory activation of platelets, neutrophils, monocytes, endothelial cells, and natural killer cells. pCRP is the substrate for mCRP, which forms when pCRP undergoes a conformational change that first produces pCRP*, an immunogenic, pentameric form, before fully dissociating to mCRP.

To highlight mechanisms of the CRP-mediated inflammatory response (**Figure 2**), we posit that circulating, sera-soluble pCRP begins to dissociate upon binding to multivalent ligands (PC, polycations, chromatin, etc.) in damaged membranes or in the presence of acidic inflammatory environments. The dissociation process, promoted by the proximity of hydrophobic membrane lipids, overcomes the Ca^2+^ stabilizing effect on pCRP and produces a transient intermediate form, pCRP^*^, which maintains the pentameric quaternary structure but displays the neoepitope of mCRP and the C1q binding site. C1q binding to pCRP* activates the classical C cascade, enhancing opsonization and phagocytosis in a localized proinflammatory response. Concurrently, to modulate global inflammatory damage, pCRP and/or mCRP binding to Factor H/CFHR4/C4bp/properdin inhibits the AP. As the pCRP → mCRP transition is irreversible, pCRP* → mCRP goes to completion upon dissociation from the membrane and initiates localized proinflammatory effects. mCRP promotes platelet aggregation, dense and alpha-granule secretion from platelets, and thromboxane A2 synthesis. mCRP also stimulates neutrophil migration, chemotaxis, and phagocytosis. mCRP activates cytokine release, particularly IL-1α, IL-1β, IL-6, IL-8, TNF-α, E-selectin, MCP-1, GRO-α and GRO-β, and promotes monocyte and EC adhesion. Finally, mCRP upregulates NK cell activity. Conversely, pCRP inhibits PAF-induced platelet aggregation and neutrophil activation and adhesion. The unique binding properties of pCRP and mCRP can be explained, in part, by differing preferences for IgG receptors, with mCRP favorably binding FcγRIIIa/b (CD16) and pCRP preferentially binding FcγRIIa (CD32).

Globally, the relationship of CRP isoforms and their contradictory bioactivities describe a mechanism of immune modulator response. Upon tissue damage or infection, while CRP levels are rising, pCRP → mCRP conversion is rapid with overall CRP function exhibiting mCRP’s proinflammatory characteristics (31). This short, localized, and potent response amplifies the acute phase of inflammation. Yet, mCRP’s pro-inflammatory properties require control to limit widespread tissue damage implying that the pCRP → mCRP conversion slows. At this point, elevated levels of pCRP are detectable in patient sera. Multiple studies predict that the lag between compromised tissue homeostasis and increased pCRP levels is 6-12-hrs, which corresponds to the mCRP pro-inflammatory activities discussed in this text (107–109).

The next chapter of CRP studies requires continued efforts into the characterization of pCRP and mCRP effects in localized immune responses to hone the diagnostic value of CRP for various pathologies. Moreover, given the distinct bioactivities of the CRP isoforms, an opportunity exists for small molecule and biologic tool and therapeutic development to elicit specific structure-function control. Currently, efforts in small molecule and biologic development have accomplished modulators of mCRP that either (1) inhibit pCRP to mCRP dissociation or (2) block mCRP binding to effector cells, respectively. Treatment with mCRP-specific antibodies (3C, 8C10) have been shown to relieve leukocyte infiltration in mouse models of rheumatoid arthritis and lupus nephritis and abrogate mCRP-induced memory loss in a mouse model of dementia (110–112). From a pharmacologic perspective, the first small molecule pCRP dissociation inhibitor, which is based on a bisphosphocholine scaffold, exhibited reduced myocardial infarction volume, cardiac dysfunction, inflammation, and mCRP deposition in rats (30, 113). On-going efforts in structural biology have mapped the pCRP – phosphocholine binding site, implicating key amino acids and a hydrophobic pocket that can drive further inhibitor development (112). While targeting mCRP offers a tantalizing opportunity for the next generation of anti-inflammatory drug development, a cautious approach towards understanding the implications of abrogating this conserved immune mechanism should be pursued. Regardless, the development of mCRP modulatory tools will be invaluable to further refine our understanding of CRP structure-function relationships.

## Author contributions

MO: Conceptualization, Formal Analysis, Investigation, Writing – original draft, Data curation, Writing – review & editing. MH: Formal Analysis, Writing – review & editing, Data curation, Investigation, Writing – original draft. AS: Data curation, Formal Analysis, Investigation, Writing – review & editing, Writing – original draft. HA: Writing – review & editing. AG: Writing – review & editing. GH: Writing – review & editing. PH: Formal Analysis, Writing – review & editing, Supervision. IR: Writing – review & editing. LP: Conceptualization, Data curation, Formal Analysis, Funding acquisition, Investigation, Project administration, Resources, Supervision, Writing – review & editing.

## References

[1] Yao Z Zhang Y Wu H. Regulation of C-reactive protein conformation in inflammation. Inflammation Res (2019) 68:815–23. doi: 10.1007/s00011-019-01269-1 31312858

[2] Dong Q Wright JR. Expression of C-reactive protein by alveolar macrophages. J Immunol (1996) 156:4815–20. doi: 10.4049/jimmunol.156.12.4815 8648129

[3] Yasojima K Schwab C McGeer EG McGeer PL. Generation of C-reactive protein and complement components in atherosclerotic plaques. Am J Pathol (2001) 158:1039–51. doi: 10.1016/S0002-9440(10)64051-5 PMC1850354 11238052

[4] Calabró P Willerson JT Yeh ET. Inflammatory cytokines stimulated C-reactive protein production by human coronary artery smooth muscle cells. Circulation (2003) 108:1930–2. doi: 10.1161/01.CIR.0000096055.62724.C5 14530191

[5] Jabs WJ Lögering BA Gerke P Kreft B Wolber EM Klinger MH. The kidney as a second site of human C-reactive protein formation in *vivo* . Eur J Immunol (2003) 33:152–61. doi: 10.1002/immu.200390018 12594844

[6] Calabro P Chang DW Willerson JT Yeh ET. Release of C-reactive protein in response to inflammatory cytokines by human adipocytes: linking obesity to vascular inflammation. J Am Coll Cardiol (2005) 46:1112–3. doi: 10.1016/j.jacc.2005.06.017 16168299

[7] Kang DH Park SK Lee IK Johnson RJ. Uric acid-induced C-reactive protein expression: implication on cell proliferation and nitric oxide production of human vascular cells. J Am Soc Nephrol (2005) 16:3553–62. doi: 10.1681/ASN.2005050572 16251237

[8] Venugopal SK Devaraj S Jialal I. Macrophage conditioned medium induces the expression of C-reactive protein in human aortic endothelial cells: potential for paracrine/autocrine effects. Am J Pathol (2005) 166:1265–71. doi: 10.1016/S0002-9440(10)62345-0 PMC1602373 15793305

[9] Haider DG Leuchten N Schaller G Gouya G Kolodjaschna J Schmetterer L. C-reactive protein is expressed and secreted by peripheral blood mononuclear cells. Clin Exp Immunol (2006) 146:533–9. doi: 10.1111/j.1365-2249.2006.03224.x PMC1810406 17100775

[10] Krupinski J Turu MM Martinez-Gonzalez J Carvajal A Juan-Babot JO Iborra E. Endogenous expression of C-reactive protein is increased in active (ulcerated noncomplicated) human carotid artery plaques. Stroke (2006) 37:1200–4. doi: 10.1161/01.STR.0000217386.37107.be 16601222

[11] Vilahur G Hernández-Vera R Molins B Casaní L Duran X Padró T. Short-term myocardial ischemia induces cardiac modified C-reactive protein expression and proinflammatory gene (cyclo-oxygenase-2, monocyte chemoattractant protein-1, and tissue factor) upregulation in peripheral blood mononuclear cells. J Thromb Haemost (2009) 7:485–93. doi: 10.1111/j.1538-7836.2008.03244.x 19036073

[12] Shrive AK Gheetham GMT Holden D Myles DAA Turnell WG Volanakis JE. Three dimensional structure of human C-reactive protein. Nat Struct Biol (1996) 3:346–54. doi: 10.1038/nsb0496-346 8599761

[13] Potempa LA Maldonado BA Laurent P Zemel ES Gewurz H. Antigenic, electrophoretic and binding alterations of human C-reactive protein modified selectively in the absence of calcium. Mol Immunol (1983) 20:1165–75. doi: 10.1016/0161-5890(83)90140-2 6656768

[14] Kresl JJ Potempa LA Anderson BE. Conversion of native oligomeric to a modified monomeric form of human C-reactive protein. Int J Biochem Cell Biol (1998) 30:1415–26. doi: 10.1016/S1357-2725(98)00078-8 9924810

[15] Potempa LA Yao ZY Ji SR Filep JG Wu Y. Solubilization and purification of recombinant modified C-reactive protein from inclusion bodies using reversible anhydride modification. Biophys Rep (2015) 1:18–33. doi: 10.1007/s41048-015-0003-2 26942216 PMC4762138

[16] Crawford JR Trial J Nambi V Hoogeveen RC Taffet GE Entman ML. Plasma levels of endothelial microparticles bearing monomeric C-reactive protein are increased in peripheral artery disease. J Cardiovasc Transl Res (2016) 9:184–93. doi: 10.1007/s12265-016-9678-0 PMC4874871 26891844

[17] Li QY Li HY Fu G Yu F Wu Y Zhao MH. Autoantibodies against C-reactive protein influence complement activation and clinical course in lupus nephritis. J Am Soc Nephrol (2017) 28:3044–54. doi: 10.1681/ASN.2016070735 PMC5619952 28566480

[18] Zhang L Li HY Li W Shen ZY Wang YD Ji SR. An ELISA assay for quantifying monomeric C-reactive protein in plasma. Front Immunol (2018) 9:511. doi: 10.3389/fimmu.2018.00511 29593741 PMC5857914

[19] Samberg NL Bray RA Gewurz H Landay AL Potempa LA. Preferential expression of neo-CRP epitopes on the surface of human peripheral blood lymphocytes. Cell Immunol (1988) 116:86–98. doi: 10.1016/0008-8749(88)90212-2 2458846

[20] Sproston NR Ashworth JJ. Role of C-reactive protein at sites of inflammation and infection. Front Immunol (2018) 9:754. doi: 10.3389/fimmu.2018.00754 29706967 PMC5908901

[21] Ji S-R Wu Y Zhu L Potempa LA Sheng F-L Lu W. Cell membranes and liposomes dissociate C-reactive protein (CRP) to form a new, biologically active structural intermediate: mCRPm. FASEB J (2007) 21:284–94. doi: 10.1096/fj.06-6722com 17116742

[22] Ullah N Wu Y. Regulation of conformational changes in C-reactive protein alters its bioactivity. Cell Biochem Biophys (2022) 80:595–608. doi: 10.1007/s12013-022-01089-x 35997934

[23] Haimovich B Ji P Ginalis E Kramer R Greco R. Phospholipase A2 enzymes regulate alpha IIb beta3-mediated, but not Fc gammaRII receptor-mediated, pp125FAK phosphorylation in platelets. Thromb Haemost (1999) 81:618–24. doi: 10.1055/s-0037-1614535 10235450

[24] Wang HW Sui SF. Dissociation and subunit rearrangement of membrane-bound human C-reactive proteins. Biochem Biophys Res Commun (2001) 288:75–9. doi: 10.1006/bbrc.2001.5733 11594754

[25] Khreiss T József L Potempa LA Filep JG. Conformational rearrangement in C-reactive protein is required for proinflammatory actions on human endothelial cells. Circulation (2004) 109:2016–22. doi: 10.1161/01.CIR.0000125527.41598.68 15051635

[26] Potempa LA Qiu WQ Stefanski A Rajab IM. Relevance of lipoproteins, membranes, and extracellular vesicles in understanding C-reactive protein biochemical structure and biological activities. Front Cardiovasc Med (2022) 9. doi: 10.3389/fcvm.2022.979461 PMC9493015 36158829

[27] Trial J Potempa LA Entman ML. The role of C-reactive protein in innate and acquired inflammation: new perspectives. Inflammation Cell Signal (2016) 3:e1409. doi: 10.14800/ics.1409 PMC5058362 27738646

[28] Habersberger J Strang F Scheichl A Htun N Bassler N Merivirta RM. Circulating microparticles generate and transport monomeric C-reactive protein in patients with myocardial infarction. Cardiovasc Res (2012) 96:64–72. doi: 10.1093/cvr/cvs237 22798388

[29] Chang M-K Binder CJ Torzewski M Witztum JL. C-reactive protein binds to both oxidized LDL and apoptotic cells through recognition of a common ligand: Phosphorylcholine of oxidized phospholipids. Proc Natl Acad Sci USA (2002) 99:13043–8. doi: 10.1073/pnas.192399699 PMC130583 12244213

[30] Thiele JR Habersberger J Braig D Schmidt Y Goerendt K Maurer V. Dissociation of pentameric to monomeric C-reactive protein localizes and aggravates inflammation: in *vivo* proof of a powerful proinflammatory mechanism and a new anti-inflammatory strategy. Circulation (2014) 130:35–50. doi: 10.1161/CIRCULATIONAHA.113.007124 24982116

[31] Rajab IM Hart PC Potempa LA. How C-reactive protein structural isoforms with distinctive bioactivities affect disease progression. Front Immunol (2020) 11:2126. doi: 10.3389/fimmu.2020.02126 33013897 PMC7511658

[32] Eisenhardt SU Habersberger J Murphy A Chen YC Woollard KJ Bassler N. Dissociation of pentameric to monomeric C-reactive protein on activated platelets localizes inflammation to atherosclerotic plaques. Circ Res (2009) 105:128–37. doi: 10.1161/CIRCRESAHA.108.190611 19520972

[33] Slevin M Matou-Nasri S Turu M Luque A Rovira N Badimon L. Modified C-reactive protein is expressed by stroke neovessels and is a potent activator of angiogenesis in *vitro* . Brain Pathol (2010) 20:151–65. doi: 10.1111/j.1750-3639.2008.00256.x PMC8094831 19170684

[34] Mihlan M Blom AM Kupreishvili K Lauer N Stelzner K Bergström F. Monomeric C-reactive protein modulates classic complement activation on necrotic cells. FASEB J (2011) 25:4198–210. doi: 10.1096/fj.11-186460 21856781

[35] Strang F Scheichl A Chen YC Wang X Htun NM Bassler N. Amyloid plaques dissociate pentameric to monomeric C-reactive protein: a novel pathomechanism driving cortical inflammation in Alzheimer's disease? Brain Pathol (2012) 22:337–46. doi: 10.1111/j.1750-3639.2011.00539.x PMC8092962 21951392

[36] Yang XW Tan Y Yu F Zhao MH. Interference of antimodified C-reactive protein autoantibodies from lupus nephritis in the biofunctions of modified C-reactive protein. Hum Immunol (2012) 73:156–63. doi: 10.1016/j.humimm.2011.12.007 22192784

[37] Braig D Kaiser B Thiele JR Bannasch H Peter K Stark GB. A conformational change of C-reactive protein in burn wounds unmasks its proinflammatory properties. Int Immunol (2014) 26:467–78. doi: 10.1093/intimm/dxu056 24844702

[38] Fiedel BA. Platelet agonist synergism by the acute phase reactant C-reactive protein. Blood (1985) 65:264–9. doi: 10.1182/blood.V65.2.264.264 2578299

[39] Fiedel BA Simpson RM Gewurz H. Activation of platelets by modified C-reactive protein. Immunology (1982) 45:439–47. PMC1555234 7061105

[40] Vigo C. Effect of C-reactive protein on platelet-activating factor-induced platelet aggregation and membrane stabilization. J Biol Chem (1985) 260:3418–22. doi: 10.1016/S0021-9258(19)83638-4 3919022

[41] Potempa LA Zeller JM Fiedel BA Kinoshita CM Gewurz H. Stimulation of human neutrophils, monocytes, and platelets by modified C-reactive protein (CRP) expressing a neoantigenic specificity. Inflammation (1988) 12:391–405. doi: 10.1007/BF00915774 2459061

[42] Boncler M Kehrel B Szewczyk R Stec-Martyna E Bednarek R Brodde M. Oxidation of C-reactive protein by hypochlorous acid leads to the formation of potent platelet activator. Int J Biol Macromol (2018) 107:2701–14. doi: 10.1016/j.ijbiomac.2017.10.159 29111269

[43] Miyazawa K Kiyono S Inoue K. Modulation of stimulus-dependent human platelet activation by C-reactive protein modified with active oxygen species. J Immunol (1988) 141:570–4. doi: 10.4049/jimmunol.141.2.570 3385211

[44] Khreiss T Jozsef L Potempa LA Filep JG. Opposing effects of C-reactive protein isoforms on shear-induced neutrophil-platelet adhesion and neutrophil aggregation in whole blood. Circulation (2004) 110:2713–20. doi: 10.1161/01.CIR.0000146846.00816.DD 15492312

[45] Shephard EG Anderson R Strachan AF Kuhn SH De Beer FC. CRP and neutrophils: functional effects and complex uptake. Clin Exp Immunol (1986) 63:718–27. PMC1577559 3708910

[46] Buchta R Fridkin M Pontet M Contessi E Scaggiante B Romeo D. Modulation of human neutrophil function by C-reactive protein. Eur J Biochem (1987) 163:141–6. doi: 10.1111/j.1432-1033.1987.tb10747.x 3028793

[47] Wilkinson PC Russell RJ Allan RB. Leucocytes and Chemotaxis. Agents Actions Suppl (1977) 61–70. doi: 10.1007/978-3-0348-7290-4_6 272837

[48] Kindmark CO. Stimulating effect of C-reactive protein on phagocytosis of various species of pathogenic bacteria. Clin Exp Immunol (1971) 8:941–8. PMC1713038 4397484

[49] Mold C Nakayama S Holzer TJ Gewurz H Du Clos TW. C-reactive protein is protective against Streptococcus pneumoniae infection in mice. J Exp Med (1981) 154:1703–8. doi: 10.1084/jem.154.5.1703 PMC2186532 7299351

[50] Mold C Edwards KM Gewurz H. Effect of C-reactive protein on the complement-mediated stimulated of human neutrophils by Streptococcus pneumoniae serotypes 3 and 6. Infect Immun (1982) 37:987–92. doi: 10.1128/iai.37.3.987-992.1982 PMC347636 7129640

[51] Holzer TJ Edwards KM Gewurz H Mold C. Binding of C-reactive protein to the pneumococcal capsule or cell wall results in differential localization of C3 and stimulation of phagocytosis. J Immunol (1984) 133:1424–30. doi: 10.4049/jimmunol.133.3.1424 6747291

[52] Tatsumi N Hashimoto K Okuda K Kyougoku T. Neutrophil chemiluminescence induced by platelet activating factor and suppressed by C-reactive protein. Clin Chim Acta (1988) 172:85–92. doi: 10.1016/0009-8981(88)90123-4 3359655

[53] Zurier RB Weissmann G Hoffstein S Kammerman S Tai HH. Mechanisms of lysosomal enzyme release from human leukocytes. II. Effects of cAMP and cGMP, autonomic agonists, and agents which affect microtubule function. J Clin Invest (1974) 53:297–309. doi: 10.1172/JCI107550 4357615 PMC301465

[54] Spirer Z Zakuth V Diamant S Stabinsky Y Fridkin M. Studies on the activity of phorbol myrystate acetate on the human polymorphonuclear leukocytes. Experientia (1979) 35:830–1. doi: 10.1007/BF01968279 467608

[55] Zeller JM Sullivan BL. C-reactive protein selectively enhances the intracellular generation of reactive oxygen products by IgG-stimulated monocytes and neutrophils. J Leukoc Biol (1992) 52:449–55. doi: 10.1002/jlb.52.4.449 1328445

[56] Khreiss T Jozsef L Potempa LA Filep JG. Loss of pentameric symmetry in C-reactive protein induces interleukin-8 secretion through peroxynitrite signaling in human neutrophils. Circ Res (2005) 97:690–7. doi: 10.1161/01.RES.0000183881.11739.CB 16123332

[57] Thiele JR Zeller J Kiefer J Braig D Kreuzaler S Lenz Y. A conformational change in C-reactive protein enhances leukocyte recruitment and reactive oxygen species generation in ischemia/reperfusion injury. Front Immunol (2018) 9:675. doi: 10.3389/fimmu.2018.00675 29713320 PMC5911593

[58] Whisler RL Proctor VK Downs EC Mortensen RF. Modulation of human monocyte chemotaxis and procoagulant activity by human C-reactive protein (CRP). Lymphokine Res (1986) 5:223–8. 3747603

[59] Zahedi K Mortensen RF. Macrophage tumoricidal activity induced by human C-reactive protein. Cancer Res (1986) 46:5077–83. 3489521

[60] Ballou SP Lozanski G. Induction of inflammatory cytokine release from cultured human monocytes by C-reactive protein. Cytokine (1992) 4:361–8. doi: 10.1016/1043-4666(92)90079-7 1420997

[61] Han KH Hong KH Park JH Ko J Kang DH Choi KJ. C-reactive protein promotes monocyte chemoattractant protein-1–mediated chemotaxis through upregulating CC chemokine receptor 2 expression in human monocytes. Circulation (2004) 109:2566–71. doi: 10.1161/01.CIR.0000131160.94926.6E 15136507

[62] Cermak J Key NS Bach RR Balla J Jacob HS Vercellotti GM. C-reactive protein induces human peripheral blood monocytes to synthesize tissue factor. Blood (1993) 82:513–20. doi: 10.1182/blood.V82.2.513.513 8329706

[63] Hanriot D Bello G Ropars A Seguin-Devaux C Poitevin G Grosjean S. C-reactive protein induces pro- and anti-inflammatory effects, including activation of the liver X receptor alpha, on human monocytes. Thromb Haemost (2008) 99:558–69. doi: 10.1160/TH07-06-0410 18327405

[64] Sproston NR El Mohtadi M Slevin M Gilmore W Ashworth JJ. The effect of C-reactive protein isoforms on nitric oxide production by U937 monocytes/macrophages. Front Immunol (2018) 9:1500. doi: 10.3389/fimmu.2018.01500 30013561 PMC6036124

[65] Zouki C Beauchamp M Baron C Filep JG. Prevention of *In vitro* neutrophil adhesion to endothelial cells through shedding of L-selectin by C-reactive protein and peptides derived from C-reactive protein. J Clin Invest (1997) 100:522–9. doi: 10.1172/JCI119561 PMC508218 9239398

[66] Zouki C Haas B Chan JS Potempa LA Filep JG. Loss of pentameric symmetry of C-reactive protein is associated with promotion of neutrophil-endothelial cell adhesion. J Immunol (2001) 167:5355–61. doi: 10.4049/jimmunol.167.9.5355 11673552

[67] Venugopal SK Devaraj S Yuhanna I Shaul P Jialal I. Demonstration that C-reactive protein decreases eNOS expression and bioactivity in human aortic endothelial cells. Circulation (2002) 106:1439–41. doi: 10.1161/01.CIR.0000033116.22237.F9 12234944

[68] Singh U Devaraj S Vasquez-Vivar J Jialal I. C-reactive protein decreases endothelial nitric oxide synthase activity via uncoupling. J Mol Cell Cardiol (2007) 43:780–91. doi: 10.1016/j.yjmcc.2007.08.015 PMC2771555 17942113

[69] Mold C Gewurz H. Inhibitory effect of C-reactive protein on alternative C pathway activation by liposomes and Streptococcus pneumoniae. J Immunol (1981) 127:2089–92. doi: 10.4049/jimmunol.127.5.2089 6913608

[70] Mold C Du Clos TW Nakayama S Edwards KM Gewurz H. C-reactive protein reactivity with complement and effects on phagocytosis. Ann N Y Acad Sci (1982) 389:251–62. doi: 10.1111/j.1749-6632.1982.tb22141.x 7046579

[71] Mold C Gewurz H Du Clos TW. Regulation of complement activation by C-reactive protein. Immunopharmacology (1999) 42:23–30. doi: 10.1016/S0162-3109(99)00007-7 10408362

[72] Baum LL James KK Glaviano RR Gewurz H. Possible role for C-reactive protein in the human natural killer cell response. J Exp Med (1983) 157:301–11. doi: 10.1084/jem.157.1.301 PMC2186896 6848619

[73] James K Baum L Adamowski C Gewurz H. C-reactive protein antigenicity on the surface of human lymphocytes. J Immunol (1983) 131:2930–4. doi: 10.4049/jimmunol.131.6.2930 6358357

[74] Bray RA Samberg NL Gewurz H Potempa LA Landay AL. C-reactive protein antigenicity on the surface of human peripheral blood lymphocytes. Characterization of lymphocytes reactive with anti-neo-CRP. J Immunol (1988) 140:4271–8. doi: 10.4049/jimmunol.140.12.4271 2453575

[75] Hamoudi WH Baum LL. Anti-C-reactive protein inhibits the calcium-dependent stage of natural killer cell activation. J Immunol (1991) 146:2873–8. doi: 10.4049/jimmunol.146.8.2873 2016530

[76] Pasceri V Willerson JT Yeh ET. Direct proinflammatory effect of C-reactive protein on human endothelial cells. Circulation (2000) 102:2165–8. doi: 10.1161/01.CIR.102.18.2165 11056086

[77] Kaplan MH Volanakis JE. Interaction of C-reactive protein complexes with the complement system. I. Consumption of human complement associated with the reaction of C-reactive protein with pneumococcal C-polysaccharide and with the choline phosphatides, lecithin and sphingomyelin. J Immunol (1974) 112:2135–47. doi: 10.4049/jimmunol.112.6.2135 4151108

[78] Siegel J Rent R Gewurz H. Interactions of C-reactive protein with the complement system. I. Protamine-induced consumption of complement in acute phase sera. J Exp Med (1974) 140:631–47. doi: 10.1084/jem.140.3.631 PMC2139624 4472155

[79] Siegel J Osmand AP Wilson MF Gewurz H. Interactions of C-reactive protein with the complement system. II. C-reactive protein-mediated consumption of complement by poly-L-lysine polymers and other polycations. J Exp Med (1975) 142:709–21. doi: 10.1084/jem.142.3.709 PMC2189923 809531

[80] Mold C Rodgers CP Richards RL Alving CR Gewurz H. Interaction of C-reactive protein with liposomes. III. Membrane requirements for binding. J Immunol (1981) 126:856–60. doi: 10.4049/jimmunol.126.3.856 7462634

[81] Volanakis JE. Complement activation by C-reactive protein complexes. Ann N Y Acad Sci (1982) 389:235–50. doi: 10.1111/j.1749-6632.1982.tb22140.x 7046577

[82] Volanakis JE Narkates AJ. Binding of human C4 to C-reactive protein-pneumococcal C-polysaccharide complexes during activation of the classical complement pathway. Mol Immunol (1983) 20:1201–7. doi: 10.1016/0161-5890(83)90143-8 6558418

[83] Robey FA Jones KD Steinberg AD. C-reactive protein mediates the solubilization of nuclear DNA by complement in *vitro* . J Exp Med (1985) 161:1344–56. doi: 10.1084/jem.161.6.1344 PMC2187628 4009117

[84] Du Clos TW. Pentraxins: structure, function, and role in inflammation. ISRN Inflammation (2013) 2013:379040. doi: 10.1155/2013/379040 24167754 PMC3791837

[85] Ji SR Wu Y Potempa LA Liang YH Zhao J. Effect of modified C-reactive protein on complement activation: a possible complement regulatory role of modified or monomeric C-reactive protein in atherosclerotic lesions. Arterioscler Thromb Vasc Biol (2006) 26:935–41. doi: 10.1161/01.ATV.0000206211.21895.73 16456095

[86] Edwards KM Gewurz H Lint TF Mold C. A role for C-reactive protein in the complement-mediated stimulation of human neutrophils by type 27 Streptococcus pneumoniae. J Immunol (1982) 128:2493–6. doi: 10.4049/jimmunol.128.6.2493 7077077

[87] Mihlan M Hebecker M Dahse HM Hälbich S Huber-Lang M Dahse R. Human complement factor H-related protein 4 binds and recruits native pentameric C-reactive protein to necrotic cells. Mol Immunol (2009) 46:335–44. doi: 10.1016/j.molimm.2008.10.029 19084272

[88] O'Flynn J van der Pol P Dixon KO Prohászka Z Daha MR van Kooten C. Monomeric C-reactive protein inhibits renal cell-directed complement activation mediated by properdin. Am J Physiol Renal Physiol (2016) 310:F1308–1316. doi: 10.1152/ajprenal.00645.2014 26984957

[89] Patel SS Thiagarajan R Willerson JT Yeh ET. Inhibition of alpha4 integrin and ICAM-1 markedly attenuate macrophage homing to atherosclerotic plaques in ApoE-deficient mice. Circulation (1998) 97:75–81. doi: 10.1161/01.CIR.97.1.75 9443434

[90] Verma S Wang CH Li SH Dumont AS Fedak PW Badiwala MV. A self-fulfilling prophecy: C-reactive protein attenuates nitric oxide production and inhibits angiogenesis. Circulation (2002) 106:913–9. doi: 10.1161/01.CIR.0000029802.88087.5E 12186793

[91] Hutchinson WL Noble GE Hawkins PN Pepys MB. The pentraxins, C-reactive protein and serum amyloid P component, are cleared and catabolized by hepatocytes in *vivo* . J Clin Invest (1994) 94:1390–6. doi: 10.1172/JCI117474 PMC295264 7929814

[92] Marnell LL Mold C Volzer MA Burlingame RW Du Clos TW. C-reactive protein binds to Fc gamma RI in transfected COS cells. J Immunol (1995) 155:2185–93. doi: 10.4049/jimmunol.155.4.2185 7636267

[93] Stein MP Edberg JC Kimberly RP Mangan EK Bharadwaj D Mold C. C-reactive protein binding to FcgammaRIIa on human monocytes and neutrophils is allele-specific. J Clin Invest (2000) 105:369–76. doi: 10.1172/JCI7817 PMC377443 10675363

[94] Heuertz RM Schneider GP Potempa LA Webster RO. Native and modified C-reactive protein bind different receptors on human neutrophils. Int J Biochem Cell Biol (2005) 37:320–35. doi: 10.1016/j.biocel.2004.07.002 15474978

[95] Ji SR Ma L Bai CJ Shi JM Li HY Potempa LA. Monomeric C-reactive protein activates endothelial cells via interaction with lipid raft microdomains. FASEB J (2009) 23:1806–16. doi: 10.1096/fj.08-116962 19136614

[96] Shephard EG van Helden PD Strauss M Böhm L De Beer FC. Functional effects of CRP binding to nuclei. Immunology (1986) 58:489–94. PMC1453455 3733149

[97] Parish WE. Features of Human Spontaneous Vasculitis Reproduced Experimentally in Animals. Effects of Antiglobulins, C-Reactive Protein and Fibrin. Paper presented at: Experimental Models of Chronic Inflammatory Diseases. Berlin, Heidelberg: Springer Berlin Heidelberg (1977).

[98] Thompson D Pepys MB Wood SP. The physiological structure of human C-reactive protein and its complex with phosphocholine. Structure (1999) 7:169–77. doi: 10.1016/S0969-2126(99)80023-9 10368284

[99] Wu Y Potempa LA El Kebir D Filep JG. C-reactive protein and inflammation: conformational changes affect function. Biol Chem (2015) 396:1181–97. doi: 10.1515/hsz-2015-0149 26040008

[100] Narkates AJ Volanakis JE. C-reactive protein binding specificities: artificial and natural phospholipid bilayers. Ann N Y Acad Sci (1982) 389:172–82. doi: 10.1111/j.1749-6632.1982.tb22135.x 7046574

[101] Haapasalo K Meri S. Regulation of the complement system by pentraxins. Front Immunol (2019) 10:1750. doi: 10.3389/fimmu.2019.01750 31428091 PMC6688104

[102] Mold C Kingzette M Gewurz H. C-reactive protein inhibits pneumococcal activation of the alternative pathway by increasing the interaction between factor H and C3b. J Immunol (1984) 133:882–5. doi: 10.4049/jimmunol.133.2.882 6234363

[103] Cheng B Lv J-M Liang Y-L Zhu L Huang X-P Li H-Y. Secretory quality control constrains functional selection-associated protein structure innovation. Commun Biol (2022) 5:268. doi: 10.1038/s42003-022-03220-3 35338247 PMC8956723

[104] Ji S-R Zhang S-H Chang Y Li H-Y Wang M-Y Lv J-M. C-reactive protein: the most familiar stranger. J Immunol (2023) 210:699–707. doi: 10.4049/jimmunol.2200831 36881905

[105] Pepys MB Hirschfield GM. C-reactive protein: a critical update. J Clin Invest (2003) 111:1805–12. doi: 10.1172/JCI200318921 PMC161431 12813013

[106] Pathak A Agrawal A. Evolution of C-reactive protein. Front Immunol (2019) 10:943. doi: 10.3389/fimmu.2019.00943 31114584 PMC6503050

[107] Kushner I Feldmann G. Control of the acute phase response. Demonstration of C-reactive protein synthesis and secretion by hepatocytes during acute inflammation in the rabbit. J Exp Med (1978) 148:466–77. doi: 10.1084/jem.148.2.466 PMC2184945 702046

[108] Colley CM Fleck A Goode AW Muller BR Myers MA. Early time course of the acute phase protein response in man. J Clin Pathol (1983) 36:203–7. doi: 10.1136/jcp.36.2.203 PMC498153 6826776

[109] Nordgreen J Munsterhjelm C Aae F Popova A Boysen P Ranheim B. The effect of lipopolysaccharide (LPS) on inflammatory markers in blood and brain and on behavior in individually-housed pigs. Physiol Behav (2018) 195:98–111. doi: 10.1016/j.physbeh.2018.07.013 30077671

[110] Fujita C Sakurai Y Yasuda Y Takada Y Huang CL Fujita M. Anti-monomeric C-reactive protein antibody ameliorates arthritis and nephritis in mice. J Immunol (2021) 207:1755–62. doi: 10.4049/jimmunol.2100349 34470853

[111] García-Lara E Aguirre S Clotet N Sawkulycz X Bartra C Almenara-Fuentes L. Antibody protection against long-term memory loss induced by monomeric C-reactive protein in a mouse model of dementia. Biomedicines (2021) 9:828. doi: 10.3390/biomedicines9070828 34356892 PMC8301488

[112] Slevin M Heidari N Azamfirei L. Monomeric C-reactive protein: current perspectives for utilization and inclusion as a prognostic indicator and therapeutic target. Front Immunol (2022) 13:866379. doi: 10.3389/fimmu.2022.866379 35309334 PMC8930844

[113] Pepys MB Hirschfield GM Tennent GA Gallimore JR Kahan MC Bellotti V. Targeting C-reactive protein for the treatment of cardiovascular disease. Nature (2006) 440:1217–21. doi: 10.1038/nature04672 16642000

